# CXCR4 Inhibition Ameliorates Severe Obliterative Pulmonary Hypertension and Accumulation of C-Kit^+^ Cells in Rats

**DOI:** 10.1371/journal.pone.0089810

**Published:** 2014-02-24

**Authors:** Daniela Farkas, Donatas Kraskauskas, Jennifer I. Drake, Aysar A. Alhussaini, Vita Kraskauskiene, Harm J. Bogaard, Carlyne D. Cool, Norbert F. Voelkel, Laszlo Farkas

**Affiliations:** 1 Victoria Johnson Center for Obstructive Lung Research, Department of Internal Medicine, Division of Respiratory Disease and Critical Care Medicine, Virginia Commonwealth University, Richmond, Virginia, United States of America; 2 Department of Pulmonary Medicine, VU University Medical Center, Amsterdam, The Netherlands; 3 Department of Pathology, University of Colorado at Denver and Health Sciences Center, Denver, Colorado, United States of America; Children's Hospital Los Angeles, United States of America

## Abstract

Successful curative treatment of severe pulmonary arterial hypertension with luminal obliteration will require a thorough understanding of the mechanism underlying the development and progression of pulmonary vascular lesions. But the cells that obliterate the pulmonary arterial lumen in severe pulmonary arterial hypertension are incompletely characterized. The goal of our study was to evaluate whether inhibition of CXC chemokine receptor 4 will prevent the accumulation of c-kit^+^ cells and severe pulmonary arterial hypertension. We detected c-kit^+­^ cells expressing endothelial (von Willebrand Factor) or smooth muscle cell/myofibroblast (α-smooth muscle actin) markers in pulmonary arterial lesions of SU5416/chronic hypoxia rats. We found increased expression of CXC chemokine ligand 12 in the lung tissue of SU5416/chronic hypoxia rats. In our prevention study, AMD3100, an inhibitor of the CXC chemokine ligand 12 receptor, CXC chemokine receptor 4, only moderately decreased pulmonary arterial obliteration and pulmonary hypertension in SU5416/chronic hypoxia animals. AMD3100 treatment reduced the number of proliferating c-kit^+^ α-smooth muscle actin^+^ cells and pulmonary arterial muscularization and did not affect c-kit^+^ von Willebrand Factor^+^ cell numbers. Both c-kit^+^ cell types expressed CXC chemokine receptor 4. In conclusion, our data demonstrate that in the SU5416/chronic hypoxia model of severe pulmonary hypertension, the CXC chemokine receptor 4-expressing c-kit^+^ α-smooth muscle actin^+^ cells contribute to pulmonary arterial muscularization. In contrast, vascular lumen obliteration by c-kit^+^ von Willebrand Factor^+^ cells is largely independent of CXC chemokine receptor 4.

## Introduction

Severe pulmonary arterial hypertension (PAH) is characterized by a lumen-obliterating pulmonary microvasculopathy and complex, multicellular plexiform lesions [1]. These vascular lesions and abnormal vessel tone lead to increased pulmonary vascular resistance and right heart failure [2]. Endothelial cell (EC) apoptosis-dependent compensatory cell overgrowth appears to be an important confounding cause of lumen obliteration in severe PAH [3], [4]. Additional factors that are likely pathobiologically relevant, are mutations in the bone morphogenic protein receptor 2 and inflammation [5]._ENREF_6 However, the nature and the origin of the phenotypically altered and proliferating cells that occlude the pulmonary vascular lumen are incompletely understood.

Progenitor cells are non-terminally differentiated cells with the potential to undergo proliferation and terminal differentiation [6], [7]. Bone marrow (BM)-derived endothelial progenitor cells (EPCs) may contribute to neoangiogenesis [8]. Stem- and progenitor cell niches, harboring EPCs, hematopoietic progenitor cells and mesenchymal stem cells have been identified in the vessel wall of the systemic circulation [9]. One way to identify progenitor cells in addition to their proliferative capacity is the use of cellular markers that are not expressed by terminally differentiated cells, such as c-kit, a tyrosine kinase receptor for stem cell factor. c-kit has been originally detected on the surface of embryonic stem cells, primitive hematopoietic cells and mast cells, and signaling *via* c-kit is important for hematopoiesis and vascular development [10], [11]. In the human lung, c-kit^+^ stem cells can repopulate airways and vessels [12] and a recent study has identified that mouse lung ECs contain a c-kit^+^ population of rare vascular endothelial stem cells that can generate functional blood vessels [13].

The accumulation of stem and progenitor cells at sites of injury requires CXC chemokine receptor 4 (CXCR4), a G-protein coupled receptor for CXC chemokine ligand 12 (CXCL12). CXCR4 is expressed on progenitor and stem cells, phagocytic cells of the innate immune system and tumor cells [14]. Signaling *via* CXCR4 is important for migration of circulating and resident cells towards a CXCL12 gradient, as well as for cell survival and proliferation [14]. Activation of the CXCL12/CXCR4 axis contributes to the repair of the ischemic myocardium [15]. CXCR4 and its ligand CXCL12 have been identified in plexiform lesions of patients with advanced PAH [15], [16]._ENREF_17 However, the potential relevance of this signaling pathway for the development of lumen-obliterating pulmonary arterial lesions remains unclear.

Inhibition of CXCR4 in chronically hypoxic mice prevents the accumulation of c-kit^+^ putative HPCs in pulmonary arteries and the development of pulmonary hypertension [17], [18]. We hypothesized that c-kit^+^ cells, including c-kit^+^ progenitor cells, accumulate in and around the lumen-occluding lesions of pulmonary arteries in severe PAH and that severe PAH and accumulation of c-kit^+^ cells depends on CXCR4.

In our study, we show the spatiotemporal localization of c-kit^+^ cells in the pulmonary vascular lesions from rats with SU5416/chronic hypoxia (SuHx)-induced angioobliterative PAH [4]. Our work extends previous experimental studies by demonstrating that some c-kit^+^ cells in the pulmonary arteries express endothelial and vascular smooth muscle cell (VSMC)/myofibroblast markers. Having found elevated CXCL12 protein levels in the lungs of SuHx animals, we examined the effects of the CXCR4 inhibitor AMD3100 in the SuHx model. We demonstrate that AMD3100 treatment prevented pulmonary arterial muscularization, but only partially reduced the pulmonary arterial obliteration. The overall result was a moderate reduction in pulmonary hypertension. CXCR4 blockade reduced the total number of c-kit^+^ cells and CXCR4^+^ cells, in particular the number of c-kit^+^ α-smooth muscle actin (α-SMA)^+^ cells, CXCR4^+^ α-SMA^+^ and proliferating c-kit^+^ α-SMA^+^ cells.

## Materials and Methods

### Animal experiments

All animal experiments were approved by the institutional animal care and utilization committee of Virginia Commonwealth University, Richmond, VA (Protocol number AM10157). This study was carried out in strict accordance with the recommendations in the Guide for the Care and Use of Laboratory Animals of the National Institutes of Health. All surgery was performed under Ketamine/Xylazine anesthesia, and all efforts were made to minimize suffering. SuHx-induced severe angioobliterative PAH was established by a single subcutaneous injection of 20 mg·kg^−1^ SU5416 at day 0 and exposure to chronic hypoxia for up to 28 days, followed by housing under normoxic conditions for experiments with a total duration of 42 days [4], [19], [20]. The animals were euthanized at the given time points by exsanguination following hemodynamic measurements [19] and blood sampling. Lung and heart were removed *en bloc,* and the right lung was snap frozen for molecular biology studies. The left lung was inflated with 0.5% low-melting agarose (20 cmH_2_O) and formalin-fixed (48 h), then paraffin-embedded for Elastica van Gieson (EvG) staining, immunohistochemistry (IHC) and immunofluorescence (IF) staining, as well as *in situ* hybridization. Naïve control animals were housed at room air. SuHx animals received 5 mg·kg^−1^·day^−1^ AMD3100 (Tocris Bioscience, Ellisville, MI) or vehicle (PBS) intraperitoneally from day 1–21. At day 21, the animals were euthanized for tissue harvest.

### Histology

Paraffin-embedded and formalin-fixed lung tissue was sectioned at a thickness of 3 µm. EvG staining was performed as published previously [21]. For vascular histomorphometry, images of EvG-stained sections were randomly taken with an AXIO imager.A1 microscope, Axiocam HRc camera and Axiovision software (all Zeiss, Jena, Germany) at 100× magnification. Each animal received a numeric code to ensure objectivity. Media thickness (MT) and external diameter (ED) were measured by an investigator blinded to the treatment groups as published before and media wall thickness (MWT) was calculated according to the formula: MWT (%)  =  [2 × (MT·ED^−1^)] × 100% [21]. Pulmonary arteries were categorized according to their size as follows: small: 25 µm<ED<50 µm, medium size: 50 µm ≤ ED<100 µm. For each animal, 30–40 pulmonary arteries were measured in two orthogonal directions using ImageJ [22].

For vWF IHC, 3 µm sections were rehydrated, followed by antigen retrieval using enzymatic digestion (proteinase K, 1:50, DAKO, Carpinteria, CA) for 6 min in PBS. Then, endogenous peroxidase (3% H_2_O_2_ in PBS) was blocked for 5 min and unspecific interactions were inhibited with 1% normal swine serum (NSS) for 15 min. The sections were incubated with the primary antibody (anti-vWF A008202, DAKO, dilution 1:5000,) in 1% NSS/PBS overnight at 4°C and with the secondary, biotin-conjugated antibody (AP132B, Millipore, Billierica, MA, dilution 1:1500) in 1%NSS/PBS for 1 h at room temperature. This was followed by 45 min incubation with HRP-conjugated Streptavidin in 1%NSS/PBS (dilution 1:200, Vector Laboratories, Burlingame, CA), counterstaining with Mayer’s Hematoxylin, dehydration and mounting.

For IF stainings, 3 µm sections were rehydrated, followed by antigen retrieval with heat in citrate buffer pH 6.0 for 20 min. Then, sections were blocked with 1% NSS/PBS for 15 min and incubated with primary antibody #1 in 1% NSS in PBS overnight at 4 °C. Sections were then incubated with the secondary antibody #1 in PBS for 4 h. For double and triple IF stainings, additional sequential incubations were performed with primary and secondary antibody #2 (and #3 for triple IF) similar to #1. Finally, the sections were counterstained with 4’,6-diamidino-2-phenylindole (DAPI) 1∶20000 for 5 min and mounted in Slow Fade Gold (both Life Technologies/Invitrogen, Carlsbad, CA). The following primary antibodies were used: α-SMA (C6198, Sigma-Aldrich, St. Louis, MO, dilution 1∶200, no secondary antibody as this antibody is directly Cy3-conjugated) or (M0851, DAKO, dilution 1∶200, no label), c-kit (LS-C78828, LifeSpan Biosciences, Seattle, WA, dilution 1∶50), CXCR4 #1 (ab2074, Abcam, Cambridge, MA, dilution 1∶50), CXCR4 #2 (ab1671, Abcam, dilution 1:25), mast cell tryptase (IMG-80250, Imgenex, San Diego, CA, dilution 1∶20), PCNA (#2586, Cell Signaling Technology, Danvers, MA, dilution 1∶100), vWF (A008202, DAKO, dilution 1∶50). The following secondary antibodies were used: A11058 (Life Technologies/Invitrogen, dilution 1∶50), A21202 (Life Technologies/Invitrogen, dilution 1∶100), A21203 (Life Technologies/Invitrogen, dilution 1∶100), A21121 (Life Technologies/Invitrogen, dilution 1∶50), A21131 (Life Technologies/Invitrogen, dilution 1∶50), A21442 (Life Technologies/Invitrogen, dilution 1:100), A21443 (Life Technologies/Invitrogen, dilution 1∶50). For all IHC and IF stainings, controls with unspecific IgG were run in parallel with each staining batch and treatment group.

### In situ hybridization

5 µm thick formalin-fixed, paraffin-embedded lung tissue sections were used for *in situ* hybridization with the QuantiGene ViewRNA ISH Tissue Assay kit (Affymetrix, Cleveland, OH) according to the manufacturer’s instructions. Custom TYPE1 QuantiGene View RNA probes were obtained from Affymetrix for rat *Kit* (Gen Bank Accession number NM_022264, region 501–1476 covered by probeset). Pretreatment was performed for 10 min at 95°C and protease digestion for 20 min at 40°C. TYPE1 probes were stained with Fast red and counterstained with Gill’s Hematoxylin. A negative control (probe omitted) was run in parallel (Figure S1). Images were taken with AXIO imager.A1 microscope, Axiocam HRc camera and Axiovision software (all Zeiss)

### Quantification of IHC and IF stainings

For the quantification of vascular obliteration in vWF-stained sections, 15 images were randomly acquired for each animal from 2 transversal sections through the left lung (one at the level of the hilum, one at least 200 µm caudal of the hilum) at a magnification of 100×, containing >80 pulmonary arteries of different calibers. The images were obtained with an AXIO imager.A1 microscope, Axiocam HRc camera and Axiovision software. Each animal received a number code to ensure objectivity. For each pulmonary artery, an investigator blinded to the treatment groups classified the degree of occlusion as none, partial or complete, and measured the ED.

For quantification of double IF stainings, red, green and blue fluorescence channel images were acquired of 10–15 randomly selected pulmonary arteries from 2 transversal sections through the left lung (one at the level of the hilum, one at least 200 µm caudal of the hilum) for each animal at a magnification of 400× with an IX70 fluorescence microscope, XM10 camera and Cellsens software (all Olympus). For triple IF stainings, 10 randomly selected pulmonary arteries from 2 transversal sections through the left lung were acquired at a magnification of 630× with a laser scanning confocal microscope using specific filters for DAPI, AlexaFluor 488, 594 and 647 (see below). The images of each animal received a unique numeric code to ensure objectivity. The number of c-kit^+^, CXCR4^+^, vWF^+^ c-kit^+^, vWF^+^ CXCR4^+^, α-SMA^+^ c-kit^+^, α-SMA^+^ CXCR4^+^, PCNA^+^ c-kit^+^, PCNA^+^ c-kit^+^ vWF^+^ and PCNA^+^ c-kit^+^ α-SMA^+^ cells per pulmonary artery (lumen/lumen-occluding cells, cells in intima, media, adventitia and perivascular infiltrate) was counted in the assembled multichannel image using the cell counter plugin of ImageJ by an investigator blinded to the treatment groups.

### Confocal microscopy

Confocal microscopy was performed with a Leica TCS SP2 laser scanning confocal microscopy system or a Zeiss LSM 700 laser scanning confocal microscope system housed in the VCU Department of Anatomy and Neurobiology Microscope Facility. If confocal imaging was done in addition to widefield microscopy, the sections used for confocal microscopy were serial sections of the ones used for widefield microscopy and quantification.

### Protein lysate preparation and Western blot

One whole right lobe was homogenized in radioimmunoprecipitation assay (RIPA) buffer (tissue to buffer ratio: 0.3 g·ml^−1^) (Sigma Aldrich) supplemented with protease- and phosphatase-inhibitors (Roche Diagnostics, Indianapolis, IN; Sigma Aldrich; New England Biolabs, Ipswich, MA). After 30 min of incubation in RIPA buffer, lung protein lysate was centrifuged at 13,000×*g* for 10 min and the supernatant stored at –80°C. Protein concentration was determined by Bio-Rad Protein Assay (Bio-Rad). The protein lysate was incubated for 10 min at 70°C in SDS loading buffer (Santa Cruz Biotechnology) and 50 µg protein was loaded into each well for SDS-PAGE. After electrophoresis, proteins were blotted onto a PVDF membrane (Bio-Rad), blocked for 1 h in 5% dry milk-PBS-0.1% Tween 20 (blocking buffer) and incubated with the respective primary antibodies overnight at 4°C in blocking buffer: cleaved caspase-3 (#9661, Cell Signaling Technologies, dilution 1:1000), CXCL12 (sc-28876, Santa Cruz, dilution 1∶200), PCNA (#2586, Cell Signaling Technologies, dilution 1∶1000), β-actin (A5441, Sigma-Aldrich, dilution 1:∶00). Secondary HRP-conjugated antibodies were applied for 1 h at room temperature in blocking buffer. The blots were developed with ECL reagent (PerkinElmer, Waltham, MA) on Genemate X-ray films (BioExpress, Kaysville, UT).

### Statistical analysis

Data are presented as mean ± SEM. The groups were compared with 2-tailed unpaired Student’s *t* test (2 groups) or 1-way ANOVA followed by a multiple comparison test according to Newman-Keuls (>2 groups). Statistics and graphs were done with GraphPad Prism 5.0 (GraphPad Software). *P*<0.05 was considered significant.

## Results

### c-kit^+^ cells accumulated in experimental angioobliterative PAH

**Figure 1:**
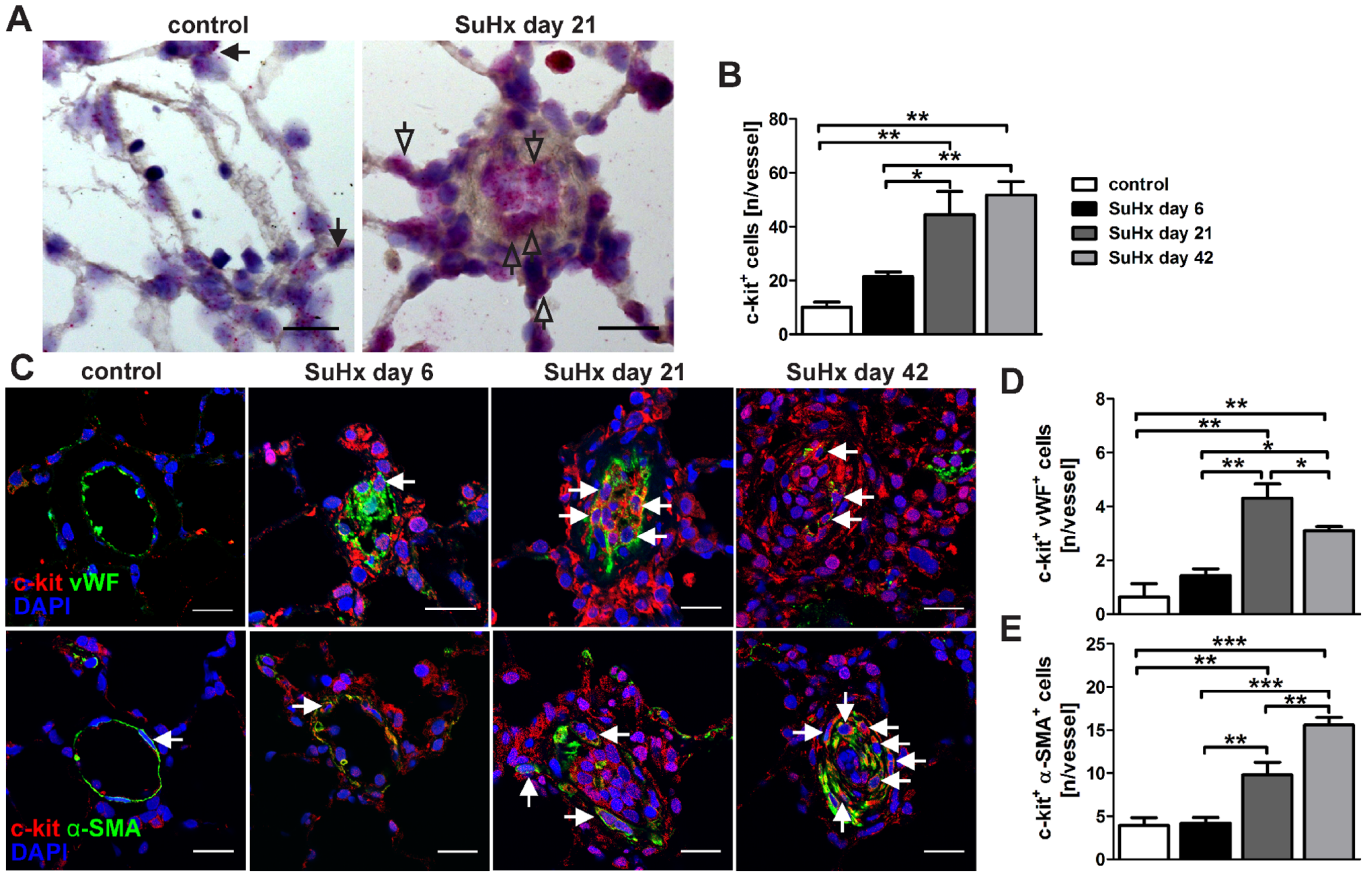
Accumulation of c-kit^+^ cells in the SU5416/chronic hypoxia (SuHx) model. (**A**) Representative images of *in situ* hybridization images demonstrating *Kit* mRNA expression. In naïve control animals, occasional low *Kit* expression was found in cells of vessels and alveolar walls**,** whereas multiple cells in lumen-obliterating regions and vessel wall/perivascular region expressed *Kit* (granular staining pattern) in the lung of a SuHx animal. The open arrows indicate cells with strong *Kit* expression. Counterstaining with Gill’s Hematoxylin. Magnification: 400×. Scale bar: 20 µm. (**B**) Quantification of the number of c-kit^+^ cells/vessel over time. n = 3 animals per group. * *P*<0.05, ** *P*<0.01. (**C**) Images demonstrate representative optical sections (confocal microscopy). c-kit^+^, c-kit^+^ von Willebrand Factor^+^ (vWF^+^) and c-kit^+^ α-smooth muscle actin^+^ (α-SMA^+^) cells were occasionally found in alveolar walls and vessel walls of naïve control animals. c-kit^+^ and c-kit^+^ α-SMA^+^ cells accumulated in and around the pulmonary arteries of SuHx animals over time. The number of c-kit^+^ vWF^+^ cells also increased until day 21 in and around the pulmonary arteries of SuHx animals and started to decline thereafter. Arrows indicate c-kit^+^ vWF^+^ or c-kit^+^ α-SMA^+^ cells. Nuclear counterstaining with 4',6-diamidino-2-phenylindole (DAPI). Magnification: 630×. Scale bar: 20 µm. (**D-E**) Quantification of the number of c-kit^+^ vWF^+^ (D) and c-kit^+^ α-SMA^+^ (E) cells per vessel. n = 3 animals per group. * *P*<0.05, ** *P*<0.01 and *** *P*<0.0001.

**Figure 2:**
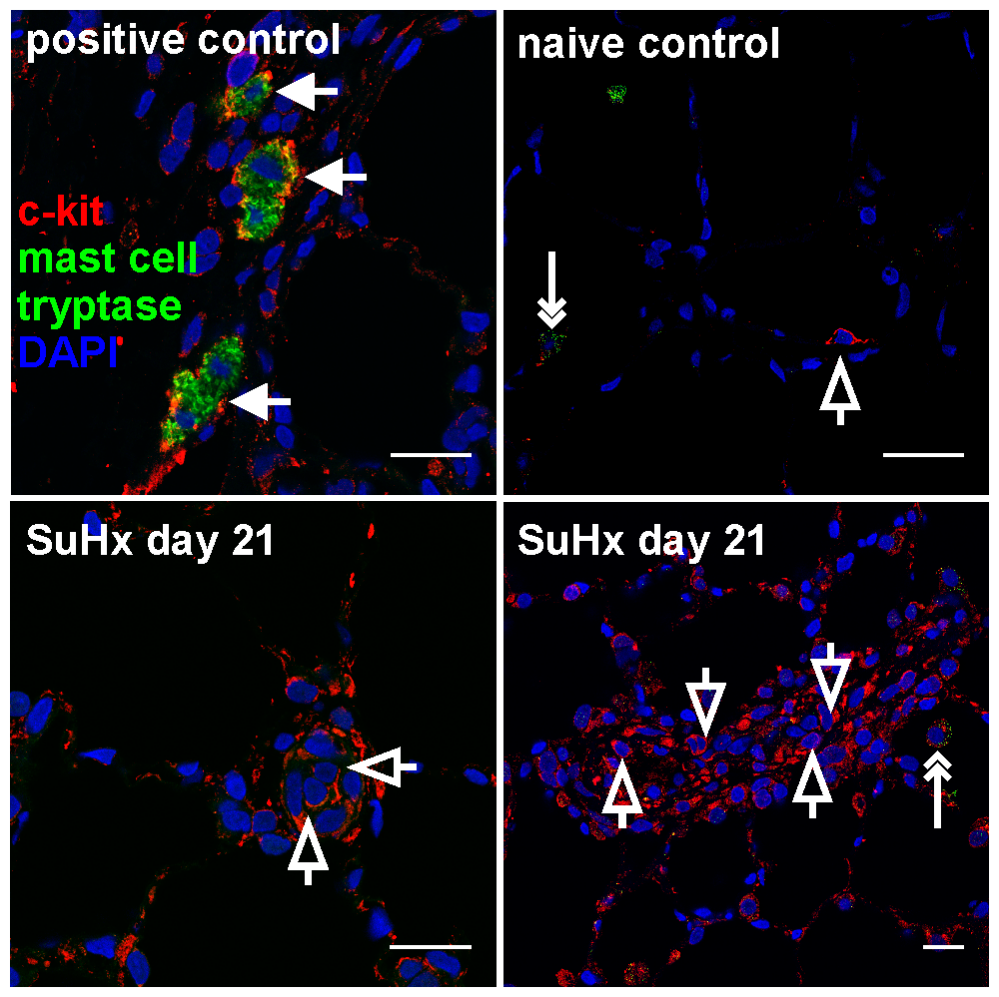
c-kit^+^ cells in the angioobliterative lesions were not mast cells. Representative optical section obtained by confocal microscopy demonstrates the presence of mast cells (c-kit^+^ mast cell tryptase^+^, arrows) around the airway mucosa of SU5416/chronic hypoxia (SuHx) animal at day 21 (positive control). In a naïve control lung, only one isolated c-kit^+^ mast cell tryptase^−^ cell was present in the pulmonary artery (open arrow). One cell with faint granular green autofluorescence (likely a macrophage) was present in the alveolar space (double-headed arrow). No mast cells were seen in the lumen-obliterating lesions of SuHx animals at day 21, only c-kit^+^ mast cell tryptase^−^ cells (open arrows). A single cell with faint granular green autofluorescence was seen in the alveolar space, likely a macrophage (double-headed arrow). Nuclear counterstaining: 4',6-diamidino-2-phenylindole (DAPI). Magnification: 630×. Scale bar: 20 µm.

First, the spatiotemporal distribution of cells expressing c-kit was examined in the lung of control and SuHx animals by *in situ* hybridization and IF staining. Then, it was investigated whether these c-kit^+^ cells express the EC marker vWF and the VSMC/myofibroblast marker α-SMA. *In-situ* hybridization and IF staining revealed that rare c-kit^+^ cells were localized in the alveolar walls and pulmonary arteries, in particular in the intima, media and adventitia layers of naïve control rats, and that some of these c-kit^+^ cells also expressed either the EC marker vWF or the VSMC/myofibroblast marker α-SMA (Figure 1). In the lungs of SuHx animals, an accumulation of c-kit^+^ cells was detected as early as day 6 and the vascular aggregates of c-kit^+^ cells were most extensive at day 21 and 42 (Figure 1). There was a progressive accumulation of c-kit^+^ vWF^+^ cells until day 21 and of c-kit^+^ α-SMA^+^ cells until day 42 (Figure 1C-E). Co-staining of c-kit with mast cell tryptase was used to investigate whether the lung vascular lesion c-kit^+^ cells were mast cells [17]. c-kit^+^ mast cell tryptase^+^ cells were not detected in and around the obliterated pulmonary arteries of SuHx animals (Figure 2).

### Lung tissue expression of CXCR4 and lung protein levels of CXCL12 in SuHx animals

**Figure 3:**
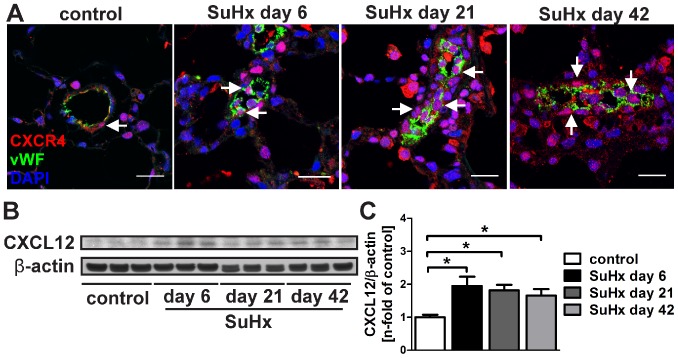
CXC chemokine receptor 4 (CXCR4) and CXC chemokine ligand 12 (CXCL12) expression. (**A**) Representative immunofluorescence stainings for CXCR4 and von Willebrand Factor (vWF) showing that while CXCR4 was found on vWF^+^ endothelial cells (arrow), perivascular cells and cells in the alveolar walls in the lungs of control animals, there was an increase in CXCR4 expression in luminal, vessel wall and perivascular cells with multiple CXCR4^+^ vWF^+^ cells (arrows) in the lumen of the pulmonary artery in the lungs of SuHx animals. Please note that the vessel shown in the image of SuHx day 6 was sectioned transversally, whereas the vessels in the images of SuHx day 21 and 42 were sectioned in a more longitudinal manner. Nuclear staining with 4',6-diamidino-2-phenylindole (DAPI). Magnification: 630×. Scale bar: 20 µm. (**B**) Representative Western blot analysis indicates that the protein expression of the CXCR4 ligand CXCL12 was increased in SuHx lung tissue protein lysate as compared to naïve control animals. β-actin was used as loading control. (**C**) Densitometry of the Western blot in (B). Densitometric values were normalized vs. β-actin and expressed as n-fold of naïve controls. n = 3 animals per group. * *P*<0.05.

In naïve control animals, CXCR4^+^ vWF^+^ ECs and CXCR4^+^ cells were identified in the pulmonary artery wall and perivascular region, as well as in the alveolar walls (Figure 3A). In SuHx animals, CXCR4^+^ cells accumulated in the lumen, pulmonary artery wall and perivascular region over time (Figure 3A). The protein expression of the CXCR4 ligand CXCL12 was increased at day 6, 21 and 42 in the lung tissue protein lysate of SuHx animals (Figure 3B-C).

### Expression of CXCR4, vWF and α-SMA in c-kit^+^ cells in SuHx animals

**Figure 4:**
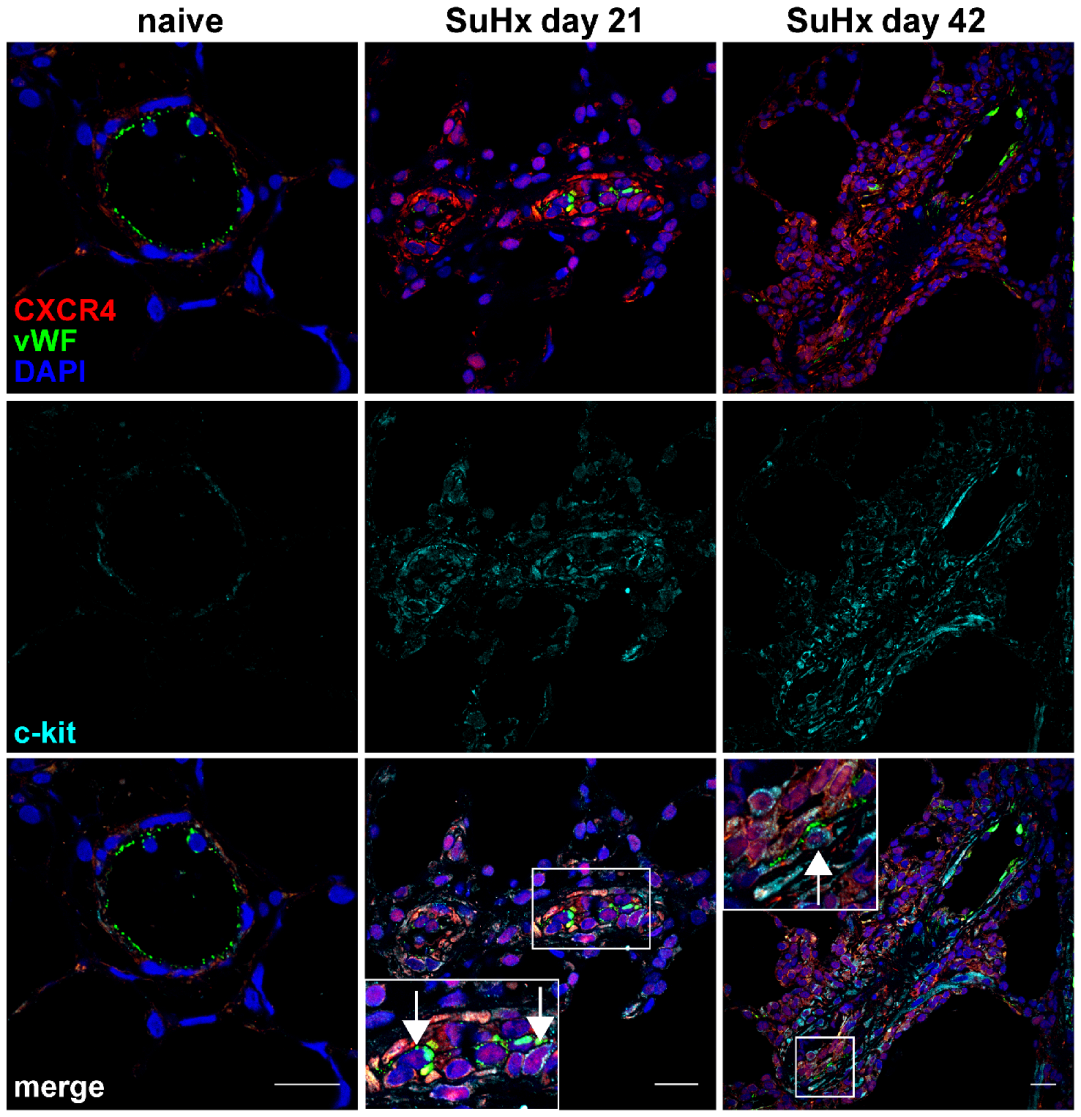
Expression of CXC chemokine receptor 4 (CXCR4) and von Willebrand Factor (vWF) in c-kit^+^ cells. Representative optical sections obtained by confocal microscopy demonstrating the co-expression of CXCR4, vWF and c-kit in the pulmonary artery wall of a naïve animal and in pulmonary vascular lesion cells of animals with SU541/chronic hypoxia (SuHx) induced severe PAH. The inserts show the area outlined by a box in more detail. Arrows indicate triple positive cells in the inserts. Nuclear counterstaining: 4’-6-diamidino-2-phenylindole (DAPI). Magnification: 400×. Scale bar: 20 µm.

**Figure 5:**
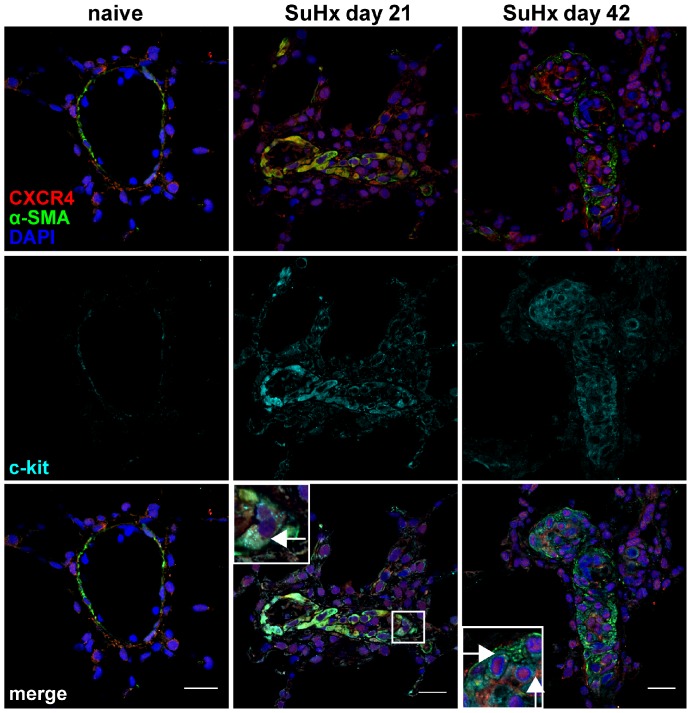
Expression of CXC chemokine receptor 4 (CXCR4) and α-smooth muscle actin (α-SMA) in c-kit^+^ cells. Representative optical sections obtained by confocal microscopy demonstrating the co-expression of CXCR4, α-SMA and c-kit in the pulmonary artery of a naïve animal and in pulmonary vascular lesion cells of animals with SU5416/chronic hypoxia (SuHx) induced severe PAH. The inserts show the area outlined by a box in more detail. Arrows indicate triple positive cells in the inserts. Nuclear counterstaining: 4′-6-diamidino-2-phenylindole (DAPI). Magnification: 400×. Scale bar: 20 µm.

In order to investigate whether various c-kit^+^ cells express CXCR4 and may therefore be a valid target for CXCR4 inhibition, the expression profile of CXCR4, vWF and α-SMA was determined in c-kit^+^ cells by triple IF stainings and confocal microscopy. The concomitant expression of CXCR4, vWF and c-kit was not detected in pulmonary arteries of naïve animals (Figure 4). In SuHx animals, multiple CXCR4^+^ vWF^+^ c-kit^+^ cells were found in pulmonary arterial lesions of SuHx animals at day 21 and 42 (Figure 4). Co-expression of the three markers CXCR4, α-SMA and c-kit was also not identified in pulmonary arteries of naïve animals (Figure 5). In contrast, multiple CXCR4^+^ α-SMA^+^ c-kit^+^ cells were detected in the obliterated pulmonary arteries of SuHx animals at day 21 and 42 (Figure 5).

### CXCR4 inhibition prevented the increased muscularization and partially the obliteration of pulmonary arteries in the SuHx model

**Figure 6:**
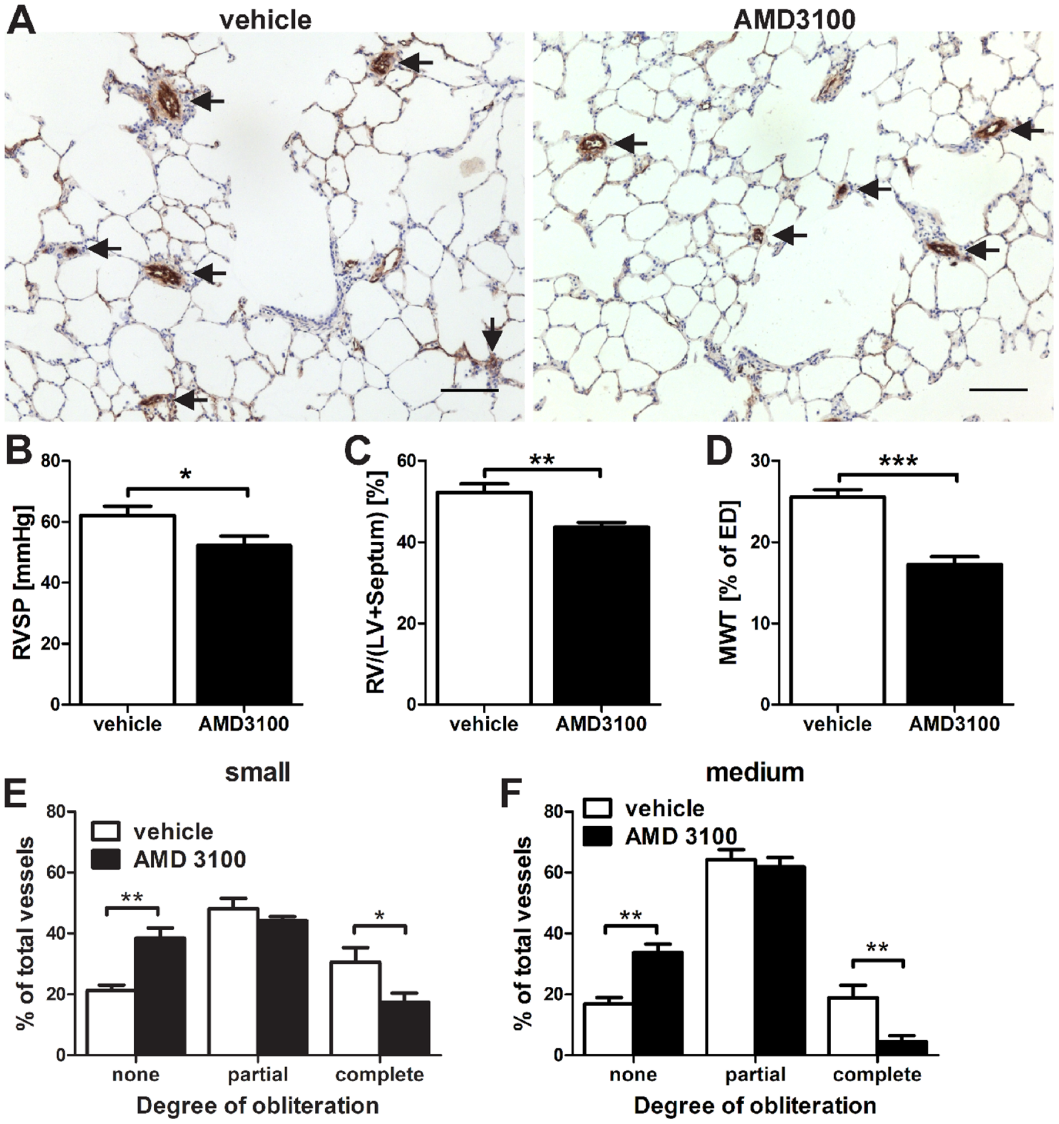
AMD3100 prevented severe pulmonary arterial hypertension (PAH) in the SU5416/chronic hypoxia (SuHx) model. (**A**) Representative von Willebrand Factor (vWF) immunohistochemistry indicates the occlusion of pulmonary arteries (arrows). These images demonstrate that treatment with the CXC chemokine receptor 4 inhibitor AMD3100 only partially prevented the obliteration of pulmonary arteries. Counterstaining: Mayer’s Hematoxylin. Magnification: 100×. Scale bar: 100 µm. (**B**) Reduced right ventricular systolic pressure (RVSP) and (**C**) decreased right ventricle (RV)/(left ventricle [LV]+Septum) ratio. (**D**) Reduced pulmonary arterial muscularization (external diameter [ED] <100 µm) was detected after AMD3100 treatment. (**E-F**) The degree of obliteration of pulmonary arteries in AMD3100-treated SuHx animals was partially reduced for small (E) (25 µm<ED<50 µm) and for medium-sized (F) (50 µm ≤ ED<100 µm) pulmonary arteries. (B-F): n = 6 animals per group. * *P*<0.05, ** *P*<0.01, *** *P*<0.0001.

To address the question whether CXCR4 signaling contributes to the lung vascular lesion formation, SuHx rats were treated with the CXCR4 inhibitor AMD3100. AMD3100 treatment moderately reduced the right ventricular (RV) systolic pressure (RVSP) and RV hypertrophy. AMD3100 treatment further completely prevented the increase in average pulmonary artery MWT, a measure of pulmonary arterial muscularization (Figure 6). CXCR4 inhibition only partially prevented the obliteration of small and medium-sized pulmonary arteries as assessed by vWF IHC (Figure 6).

### CXCR4 inhibition reduced lung cell proliferation and decreased the presence of c-kit^+^ cells

**Figure 7:**
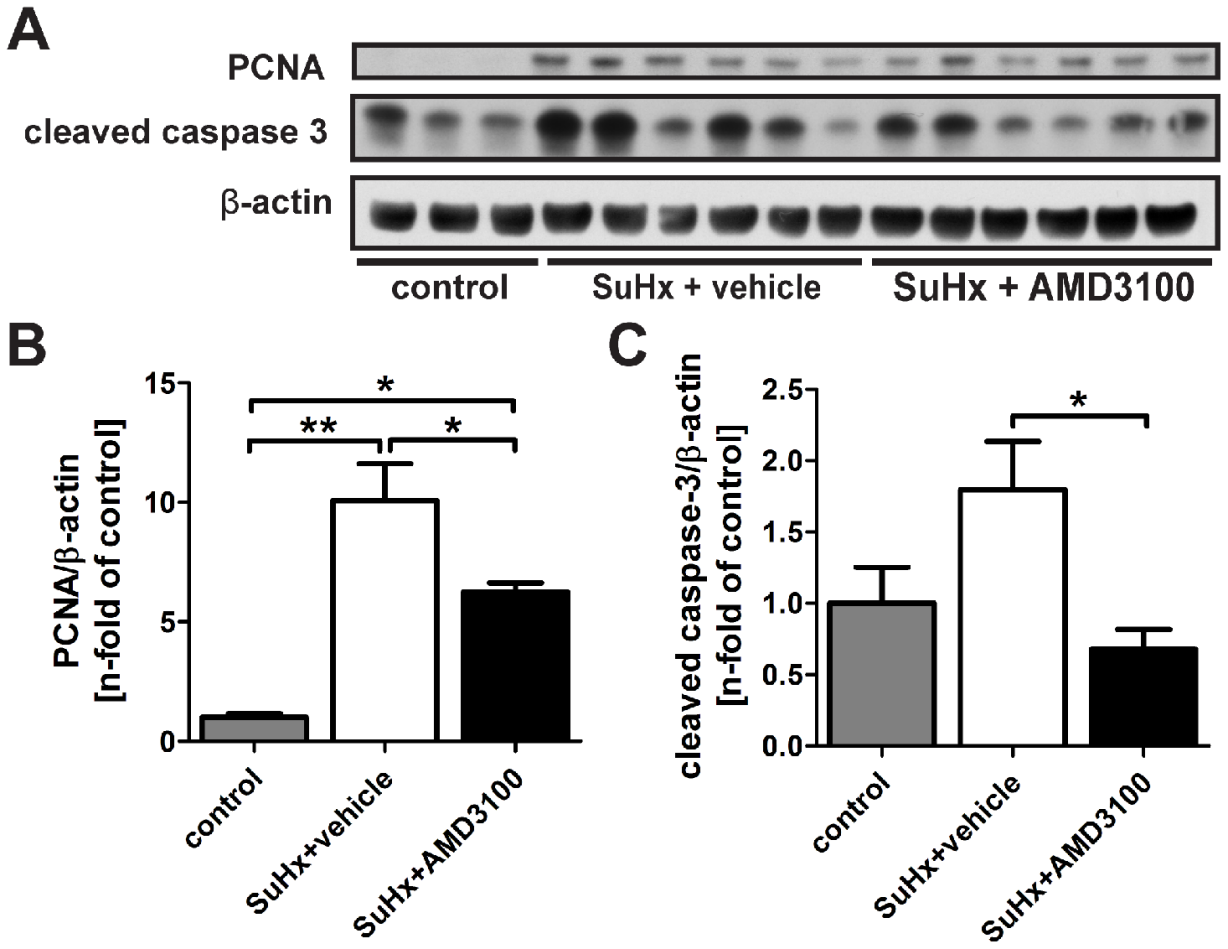
Proliferation and apoptosis in the lungs of AMD3100-treated SU5416/chronic hypoxia (SuHx) animals. (**A**) Representative Western blot for proliferating cell nuclear antigen (PCNA) and cleaved caspase-3 in the lung tissue protein lysate of naïve control animals (n = 3), SuHx + vehicle (n = 6) and SuHx + AMD3100 treated rats (n = 6). β-actin was used as loading control. (**B-C**) Densitometric analysis indicates increased PCNA (B) and cleaved caspase-3 (C) protein levels in the lungs of SuHx + vehicle animals vs. controls, and that AMD3100 treatment significantly reduced PCNA and cleaved caspase-3 protein levels in the lungs of SuHx animals. Densitometric values were normalized vs. β-actin and expressed as n-fold of naïve controls. n = 3 animals per group for controls and n = 6 animals per group for SuHx + vehicle and SuHx + AMD3100 groups. * *P*<0.05 and ** *P*<0.01.

**Figure 8:**
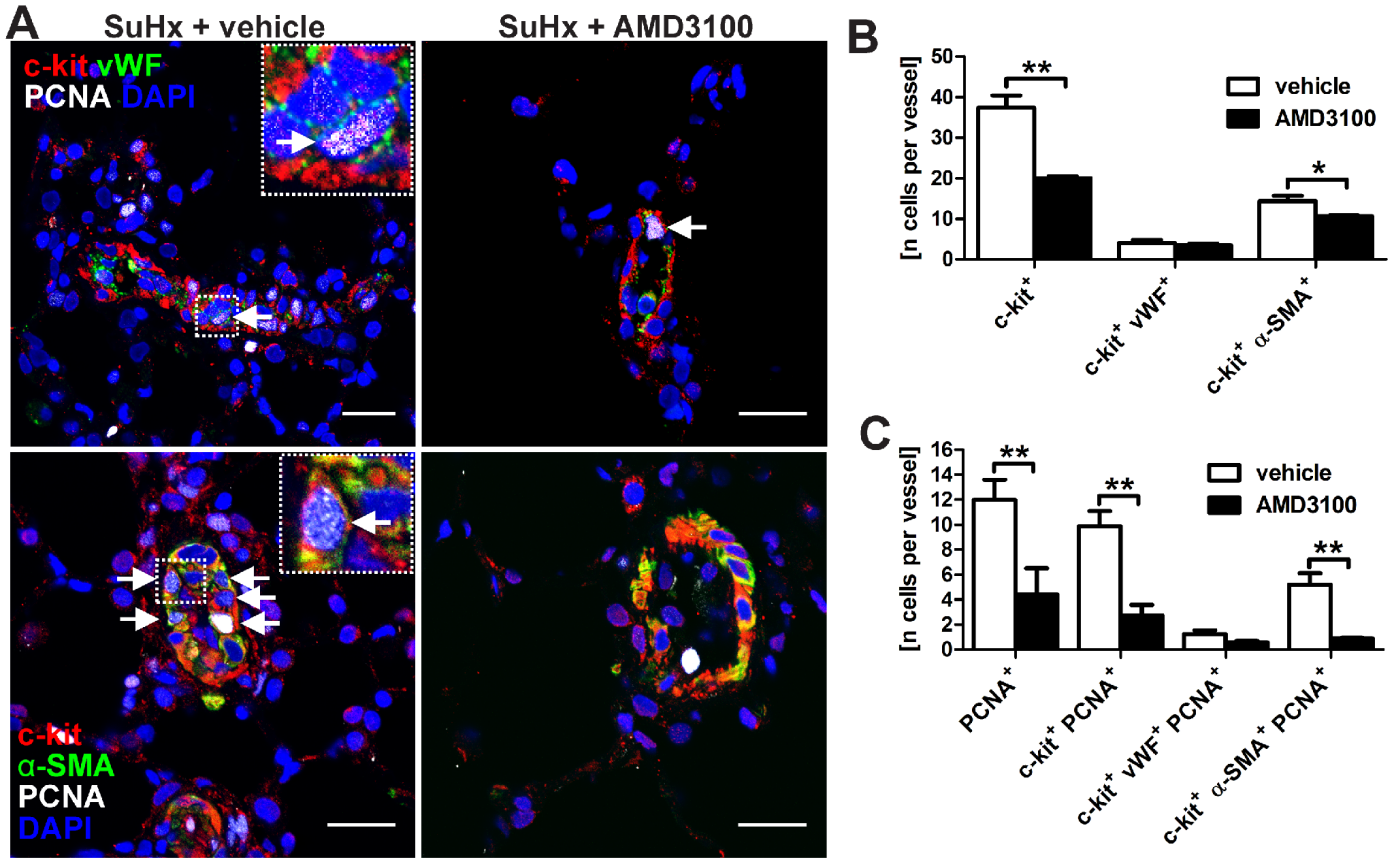
Proliferation of c-kit^+^ cells in the lungs of AMD3100-treated SU5416/chronic hypoxia (SuHx) animals. (**A**) Representative optical sections (confocal microscopy) demonstrate c-kit^+^ von Willebrand Factor^+^ (vWF^+^) proliferating cell nuclear antigen^+^ (PCNA^+^) cells (upper row) or c-kit^+^ α-smooth muscle actin^+^ (α-SMA^+^) PCNA^+^ cells (lower row) in pulmonary arteries of SuHx + vehicle and SuHx + AMD3100 treated animals. The inserts show a triple positive cell (arrow) in more detail. Please note that the bright white stained dots indicate cells with very strong PCNA staining. Nuclear counterstaining: 4',6-diamidino-2-phenylindole (DAPI). Magnification: 630×. Scale bar: 20 µm. (**B-C**) Quantification of the number of c-kit^+^, c-kit^+^ vWF^+^ and c-kit^+^ α-SMA^+^, as well as PCNA^+^, c-kit^+^ PCNA^+^, c-kit^+^ vWF^+^ PCNA^+^ and c-kit^+^ α-SMA^+^ PCNA^+^ cells in and around the pulmonary arteries of SuHx + vehicle and SuHx + AMD3100 animals. n = 3 animals per group. * *P*<0.05 and ** *P*<0.01.

AMD3100 treatment significantly reduced the protein expression of PCNA and cleaved caspase-3 in the lung tissue protein lysate of SuHx animals (Figure 7A-C). These findings were associated with the decreased presence of c-kit^+^ cells and c-kit^+^ α-SMA^+^ cells in and around pulmonary arteries, but not with a reduction in the number of c-kit^+^ vWF^+^ cells in pulmonary arteries (Figure 8A-B). Less PCNA^+^ cells, c-kit^+^ PCNA^+^ cells and c-kit^+^ α-SMA^+^ PCNA^+^ were found in and around pulmonary arteries following AMD3100 treatment, but a significant reduction in the number of c-kit^+^ vWF^+^ PCNA^+^ cells was not detected (Figure 8A, C). These results indicate that the number and the proliferation of these c-kit^+^ vWF^+^ cells were not affected by CXCR4 inhibition. Hence, the reduced proliferation of primitive α-SMA^+^ c-kit^+^ cells after AMD3100 treatment correlated with the prevention of increased muscularization in pulmonary arteries.

### The accumulation of vWF^+^ CXCR4^+^ cells was not significantly reduced by AMD3100

**Figure 9:**
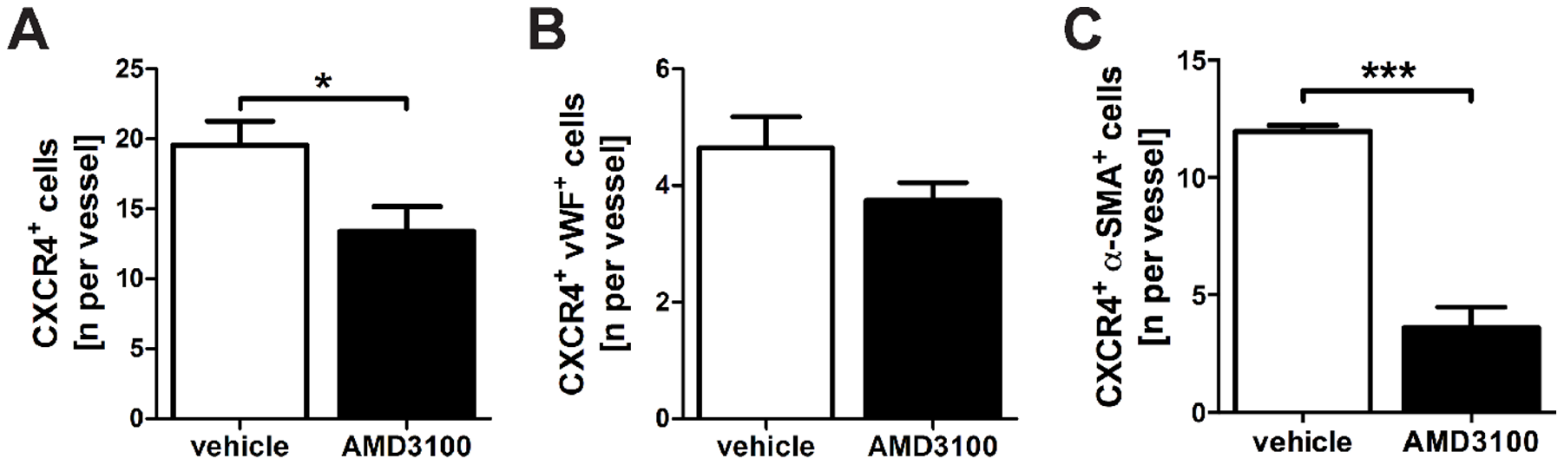
Effect of AMD3100 treatment on CXC chemokine receptor 4^+^ (CXCR4^+^) cells. Quantification of the number of total CXCR4^+^ cells (**A**), CXCR4^+^ von Willebrand Factor^+^ (vWF^+^) cells (**B**) and CXCR4^+^ α-smooth muscle actin^+^ (α-SMA^+^) cells (**C**) per vessel in pulmonary arteries of SuHx animals treated with vehicle or AMD3100. The data indicate that while the number of total CXCR4^+^ cells and CXCR4^+^ α-SMA^+^ cells per vessel was significantly reduced by AMD3100 treatment, there was only a small trend towards decreased number of CXCR4^+^ vWF^+^ cells per vessel in AMD3100 treated SuHx animals. n = 4 animals/group. * *P*<0.05, *** *P*<0.0001.

AMD3100 treatment reduced the total number of CXCR4^+^ cells and CXCR4^+^ α-SMA^+^ per vessel in the pulmonary arteries of SuHx animals, but there was only a small trend towards reduced number of CXCR4^+^ vWF^+^ cells after AMD3100 treatment (Figure 9A-C).

## Discussion

The pathophysiology of severe PAH has been traditionally explained largely as a consequence of chronic hemodynamic stress, such as pulmonary vasoconstriction and elevated shear stress [23]. The increase in the pulmonary vascular resistance is now also attributed to the structural alterations of the pulmonary arteries and reversal of these structural changes has become a treatment goal [2]. Successful and permanent reversal of the pulmonary arterial remodeling will likely require a detailed knowledge of the altered microenvironment of the remodeled pulmonary artery [2]. The early phase of lung vessel disease development in human PAH remains unexamined and it is not feasible to serially study diseased human lungs. Hence, we investigated the development of vascular obliteration in an animal model of severe PAH that predictably generates obliterative pulmonary arterial lesions resembling those observed in the lungs of patients with severe PAH [4], [24].

The pulmonary vascular lesions in severe PAH are complex and multicellular, and it has been suggested that the lesions contain primitive angiogenic cells that express endothelial markers [25]. Indeed, a variety of stem and progenitor cell markers has been detected in vascular lesions of patients with severe PAH [16], [17], [26]. BM-derived progenitors from PAH patients can induce vascular remodeling, including *in situ* thrombi, in the pulmonary arteries of mice [27]._ENREF_15_ENREF_30 In the present study, we concentrated on the role of cells expressing the tyrosine kinase receptor c-kit: c-kit is a common marker of primitive progenitor and stem cells, including the recently described lung stem cells [12] and rare vascular endothelial stem cells [13]. c-kit^+^ mast cells and c-kit^+^ progenitor cells have also been identified in pulmonary vascular lesions of patients with severe PAH [17], [26] and around pulmonary arteries of chronic hypoxic mice [18].

The main findings of our experimental studies are: 1) c-kit^+^ cells accumulate in and around the pulmonary artery lesions in the SuHx model of severe obliterative PAH. 2) Some c-kit^+^ cells express endothelial and mesenchymal markers. 3) CXCR4^+^ cells accumulate in the pulmonary vascular lesions of SuHx animals and the CXCR4 ligand CXCL12 is found at elevated protein levels in the lung tissue of SuHx animals. 4) c-kit^+^ cells in the pulmonary vascular lesions frequently express CXCR4. 5) CXCR4 blockade affects c-kit^+^ cell participation, in particular the proliferation of α-SMA^+^ c-kit^+^ cells, and pulmonary arterial muscularization. 6) Occlusion of pulmonary arteries and severe PAH were only partially prevented by CXCR4 blockade. 7) CXCR4^+^ α-SMA^+^ cells, but not CXCR4^+^ vWF^+^ cells, were reactive to AMD3100 treatment.

Similar to human PAH [17], [26], we identified in our study in the lungs of the animals with severe SuHx-induced PAH c-kit^+^ cells within the lumen-occluding cells and in the perivascular cellular aggregates. We extend the current knowledge of the presence of c-kit^+^ cells in pulmonary vascular lesions by demonstrating that the c-kit^+^ cells found in the pulmonary vascular lesions are not a uniform population: Some c-kit^+^ cells in the pulmonary arterial wall expressed the endothelial marker vWF or the VSMC/myofibroblast marker α-SMA. c-kit expression has been demonstrated on a variety of cell types, including mast cells, dendritic cells, pre-T and pre-B lymphocytes, hematopoietic progenitor and stem cells, as well as various forms of tissue-derived progenitor and stem cells [12], [13], [17], [28]. As expected, a small number of c-kit^+^ cells were also detected in the pulmonary arteries and alveolar walls from control animals. The population of c-kit^+^ cells, and in particular the pool of c-kit^+^ vWF^+^ and c-kit^+^ α-SMA^+^ cells, was vastly expanded in and around the pulmonary arterial lesions of SuHx animals. It is interesting that the presence of c-kit^+^ vWF^+^ cells started to decline after day 21 in the SuHx model. At the same time, c-kit^+^ α-SMA^+^ cell continued to accumulate in the remodeled pulmonary arteries and were also increasingly found among lumen-obliterating cells. One possible explanation is the transdifferentiation of c-kit^+^ EPCs into α-SMA expressing VSMC or myofibroblast precursors in more mature lesions, a process that has been already suggested *in vitro* [29]-[31]. Because α-SMA expression is also frequently found in activated myofibroblasts in scar tissue, the c-kit^+^ α-SMA^+^ may also represent myofibroblast precursors [32], [33] that accumulate in the obliterated pulmonary arteries during a process of intravascular scar formation. Fibrocytes, which are characterized as cells expressing hematopoietic and mesenchymal markers, have been identified as an important source of myofibroblasts in the context of lung fibrosis [34]-[36]. Because fibrocytes express markers of immature hematopoietic cells, such as CD34, it is possible that at least a fraction of the c-kit^+^ cells, the c-kit^+^ α-SMA^+^ cells, represents fibrocytes [34], [35]. Fibrocytes have been suggested to contribute to pulmonary vascular remodeling in mice, rats and calves under conditions of chronic hypoxia [33], [37] and fibrocytes may also contribute to pulmonary vascular remodeling in severe obliterative PAH.

The receptor for CXCL12, CXCR4, is important for the homing of stem and progenitor cells to sites of tissue injury [38]. CXCR4 is expressed in the lung vascular lesions of patients with severe PAH [16], [26]. Therefore, we investigated the expression of CXCR4 and CXCL12 in the lungs of SuHx animals. We found CXCR4 expression in the cells that accumulated over time in the lumen, pulmonary artery wall and perivascular region of SuHx animals. CXCR4 expression was also frequently detected in c-kit^+^ cells, including the c-kit^+^ vWF^+^ and c-kit^+^ α-SMA^+^ cells that clustered in and around the remodeled pulmonary arteries of SuHx animals. Because the accumulation of CXCR4^+^ cells is directed by the tissue expression of its ligand CXCL12 [39], it is not surprising that the expression of the CXCR4 ligand CXCL12 was significantly increased in the lung tissue of SuHx animals. The expression of CXCL12 is extensively regulated by tissue hypoxia and by local tissue injury [36], [40]. We suggest that the upregulation of CXCL12 in the lungs of SuHx animals is likely dependent on chronic hypoxia, as demonstrated in chronic hypoxic mice [18], [41], and the ongoing vascular injury originally initiated by injection of SU5416 [3], [29]. The increased expression of CXCL12, the accumulation of CXCR4^+^ cells and the expression of CXCR4 in the c-kit^+^ cells in the lung vascular lesions provided the rationale to investigate whether the formation of lesions in the pulmonary arteries was dependent on CXCL12/CXCR4.

CXCR4 inhibition with AMD3100 reduced the accumulation and proliferation of c-kit^+^ cells and the muscularization of the remodeled pulmonary arteries from SuHx animals. The main effect was decreased proliferation of c-kit^+^ α-SMA^+^ cells, a finding that provides a cellular correlate to the morphologically reduced muscularization of pulmonary arteries. Our findings in a rat model of severe, obliterative PAH complement recent work in chronic hypoxic mice indicating that c-kit^+^ cells may contribute to pulmonary arterial muscularization in a CXCR4-dependent manner [18]. The findings that c-kit^+^ α-SMA^+^ cells express CXCR4 and accumulate in a CXCR4-dependent manner in the remodeled pulmonary arteries further support the concept that these c-kit^+^ α-SMA^+^ CXCR4^+^ cells may be fibrocytes that contribute to pulmonary vascular remodeling. Various studies have shown that CXCR4 inhibition reduces lung fibrosis *via* reduced fibrocyte migration [36], [42], [43]. However, in SuHx animals, CXCR4 inhibition only caused a modest reduction in PAH and did not significantly decrease the number of c-kit^+^ vWF^+^ or proliferating c-kit^+^ vWF^+^ cells in pulmonary arteries. Our data also indicate that the accumulation of CXCR4^+^ vWF^+^ ECs/EPCs is largely independent of CXCR4, in contrast to the accumulation of CXCR4^+^ cells and CXCR4^+^ α-SMA^+^ cells. We propose that the cells retained in the obliterated pulmonary arteries may represent a c-kit^+^ EPC pool, that was largely unaffected by AMD3100. We base our assumption on the localization of the cells in the lumen obliterating lesions and the co-expression of the EC marker vWF with c-kit in these cells [13]. It is possible that CXCR4 inhibition may not only decrease homing of circulating cells, but may also reduce migration of lung resident cells or impact the function of stem/progenitor cells [44], [45]._ENREF_56 Because AMD3100 does not affect the c-kit^+^ vWF^+^ cells, we propose that these c-kit^+^ vWF^+^ cells are likely lung resident EPCs and/or that their migration and homing is not critically dependent on CXCR4 [13], [18], [29], [36], [46]. Our data indicate that lumen obliteration by vWF^+^ cells may be promoted by mechanisms in addition to CXCL12/CXCR4 signaling. One example of such an additional mechanism may be the increased expression of the pro-angiogenic factor fibroblast growth factor 2. Fibroblast growth factor 2 has been shown to be elevated in pulmonary arterial lesions of PAH patients and animals with SuHx-induced severe PAH [47], [48].

The reduction in RVSP following CXCR4 inhibition was only moderate in SuHx rats – in contrast to the studies conducted in the chronic hypoxic model of PAH. Our data suggest that the persistent RVSP elevation can be explained, similar to concepts in human severe PAH, by the residual obliteration of pulmonary arteries that was only partially prevented by CXCR4 blockade [2]. The data further indicate that the arteriolar occlusion is due to a different set of c-kit^+^ cells, likely EPCs, that seem to be largely unaffected by AMD3100 treatment.

In conclusion, our data indicate that c-kit^+^ progenitor cells accumulate in and around the lung vascular lesions of SuHx rats. Our data also suggest that in severe experimental PAH, lung vascular obliteration and the accumulation of c-kit^+^ cells only partially depend on CXCR4 activity. In contrast, CXCR4 signaling is required for pulmonary arterial muscularization. c-kit^+^ α-SMA^+^ cells appear to contribute to pulmonary arterial muscularization in a CXCR4-dependent manner. A different subset of c-kit^+^ cells with endothelial markers seems to contribute to pulmonary arterial obliteration in a largely CXCR4-independent fashion. Whether c-kit^+^ progenitor cell populations are sufficient to cause obliterative pulmonary vascular disease or whether additional factors are required will have to be examined by cell transfer experiments.

## Supporting Information

Figure S1 **Negative control for** ***in situ*** **hybridization.** Negative control (SU5416/chronic hypoxia angioobliterative lesion at day 21) was generated by omitting the hybridization probe. Counterstaining with Gill’s Hematoxylin. Magnification: 400×. Scale bar: 20 µm.
(TIF)
