# Supplementary figures and images for: CXCR4 Inhibition Ameliorates Severe Obliterative Pulmonary Hypertension and Accumulation of C-Kit+ Cells in Rats
Source: PLoS One. 2014 Feb 24;9(2):e89810. doi: 10.1371/journal.pone.0089810 (PMC3933653; doi:10.1371/journal.pone.0089810)

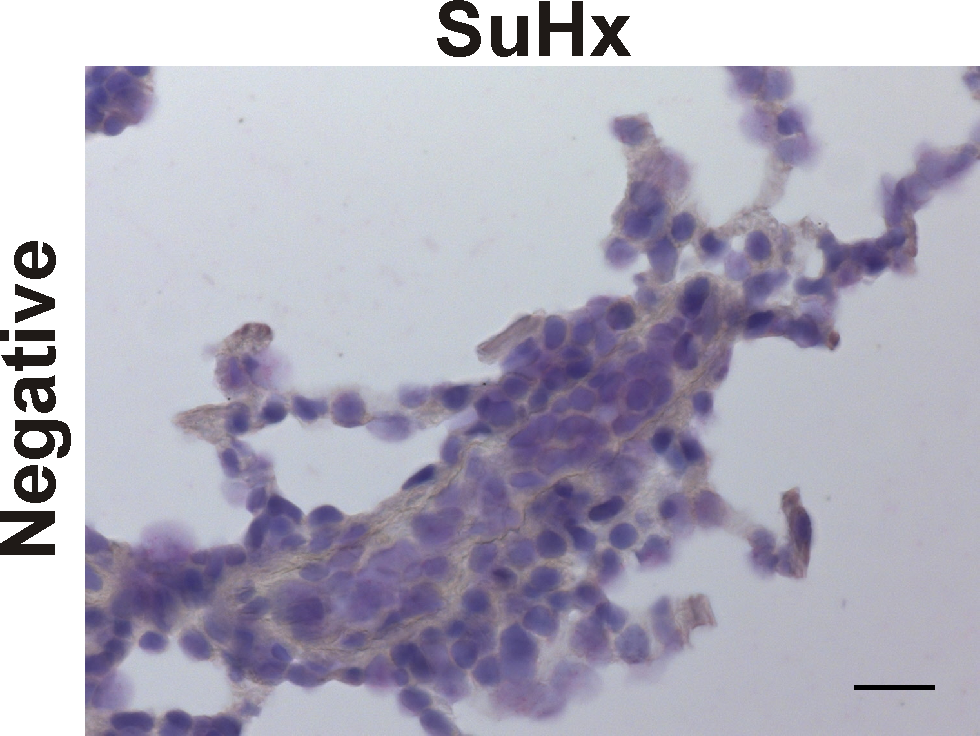

Supplement: Figure S1 — Negative control for in situ hybridization. Negative control (SU5416/chronic hypoxia angioobliterative lesion at day 21) was generated by omitting the hybridization probe. Counterstaining with Gill’s Hematoxylin. Magnification: 400×. Scale bar: 20 µm. (TIF) [file pone.0089810.s001.tif]
